# Role of Probiotics in Short Bowel Syndrome in Infants and Children—A Systematic Review

**DOI:** 10.3390/nu5030679

**Published:** 2013-03-05

**Authors:** Vudum S. Reddy, Sanjay K. Patole, Shripada Rao

**Affiliations:** 1 Department of Neonatology, King Edward Memorial Hospital for Women, Subiaco, Perth, WA 6008, Australia; E-Mail: vudum.reddy@health.wa.gov.au; 2 Centre for Neonatal Research and Education, University of Western Australia, Perth, WA 6008, Australia; E-Mail: shripada.rao@health.wa.gov.au; 3 Department of Neonatology, Princess Margaret Hospital, Perth, WA 6008, Australia

**Keywords:** infants, children, probiotics, review, short bowel syndrome

## Abstract

Short bowel syndrome (SBS) is a cause of significant morbidity and mortality in children. Probiotics, due to their beneficial effects on the gastrointestinal tract (e.g., improving gut barrier function, motility, facilitation of intestinal adaptation and decreasing pathogen load and inflammation) may have a therapeutic role in the management of SBS. To conduct a systematic review of the current evidence for the effects of probiotic supplementation in children with SBS, the standard Cochrane methodology for systematic reviews was used. The databases, Pubmed, Embase, ACTR, CENTRAL, and the international trial registry, and reference lists of articles were searched for randomised (RCT) or quasi-randomised controlled trials reporting on the use of probiotics in SBS. Our search revealed no RCTs on the use of probiotics in children with SBS. We found one small cross-over RCT (placebo controlled crossover clinical trial), one case control study and nine case reports on the use of probiotics in children with SBS. In the crossover RCT, there was no consistent effect on intestinal permeability (primary outcome) after supplementation with *Lactobacillus rhamnosus* (LGG) in nine children with SBS. The case control study (four cases: four controls) reported a trend for increase in height and weight velocity and improvement in non-clinical outcomes, such as gut flora, lymphocyte count and serum prealbumin. Five of the nine case reports showed that children (*n* = 12) with SBS were benefited (e.g., cessation of diarrhoea, improved faecal flora, weight gain and weaning from parenteral nutrition) by probiotic supplementation. The remaining four reported on the adverse effects, such as *Lactobacillus* sepsis (*n* = 3) and D-lactic acidosis (*n* = 2). There is insufficient evidence on the effects of probiotics in children with SBS. The safety and efficacy of probiotic supplementation in this high-risk cohort needs to be evaluated in large definitive trials.

## 1. Introduction

Intestinal failure (IF) has been defined as the critical reduction of functional gut mass below the minimal amount necessary for adequate digestion and absorption to satisfy body nutrient and fluid requirements for maintenance in adults or growth in children [1]. Short-bowel syndrome (SBS) is the most common cause of IF in infants; other causes being motility disorders (aganglionosis), chronic intestinal pseudo-obstruction syndrome (myopathic and neuropathic) and congenital diseases of enterocyte development [1]. SBS results from surgical resection, congenital defect or disease-associated loss of absorption capacity of the gut and is characterized by the inability to maintain protein-energy, fluid, electrolyte or micronutrient balances when on a conventionally accepted, normal diet [2]. These patients are therefore dependent on parenteral nutrition (PN). The duration of PN significantly correlates with the length of residual gut [3]. SBS has also been defined as the need for PN greater than 42 days or 2 mo after bowel resection of ≥70% or a residual small bowel length of less than 25% of that expected for gestational age [4,5].

The most common cause of SBS in the neonatal period is necrotizing enterocolitis (NEC), accounting for 35%–50% of cases [6,7]. The other causes include abdominal wall defects (gastroschisis, omphalocele), midgut volvulus, intestinal atresia, meconium ileus, Hirschsprung’s disease and superior mesenteric artery abnormalities [6,8]. The contribution of NEC to SBS appears to be decreasing in some centres, due to advances in perinatal care and antenatal steroids, resulting in the decreased incidence of NEC [5,9]. Neonatal research network hospitals in the US have reported an incidence of 7/1000 in very low birth weight (VLBW) infants and 11/1000 in extremely low birth weight (ELBW) infants [10]. Similar to NEC, birth weight and gestational age were inversely related to the incidence of SBS. NEC was responsible for 96% of SBS cases. In a Canadian study, the incidence was estimated to be 22.1 per 1000 NICU admissions at a tertiary centre, whereas population-based incidence was 24.5 per 100,000 live births; only three out of 40 SBS infants were of term gestation [11]. An Italian study reported an incidence of 5/1000 NICU admissions and 1/1000 live births [9]. Approximately 80% of SBS in the paediatric population occurs in the neonatal period [8].

The health burden of SBS is significant. A case fatality rate of 27.5%–37.5% has been reported within 1.5–5 year follow-up periods in four retrospective studies, and hepatic failure accounted for 60% and sepsis for 10%–20% of deaths [5,6,7,12]. Incidence of sepsis is high and is the most common cause for readmission in patients with SBS, increasing the length of hospitalization and the cost of care [5,10,13]. Growth deficits (weight, length and head circumference) were prevalent in 74% of VLBW infants with SBS at 18–22 month age [10]. Failure to thrive (body weight < fifth percentile) was seen in 76.5% of patients at 6 mo and in 47.6% at 2.5 year in a retrospective study [7]. SBS imposes disproportionately high healthcare costs on tax payers. In the United States, the mean total cost of care per child over a five-year period after onset of SBS was estimated to be over 1.62 million (range 1.3–2 million) USD, of which hospitalization accounted for the maximum cost [5]. Shorter residual bowel length could incur higher costs. PN dependence ranged from 2.4 months to 12.6 years, with a median of 1.5 years. In the Netherlands, the average total cost was 355,000 USD, with a maximum of 600,000 USD [14].

## 2. Post-Resection Changes and Complications in SBS

### 2.1. Intestinal Adaptation

The key to successful weaning from PN in SBS is small bowel adaptation. The process by which the residual bowel increases its absorptive surface area and functional capacity to meet the body’s metabolic and growth needs is called adaptation [15]. There is an increase in length, thickness and circumference of the bowel, villus height, depth of crypts, rate of enterocyte proliferation, the number of epithelial cells per villus, activity of enzymes and the rate of absorption per cm of intestine [15,16]. Enteral nutrition is the single most important factor contributing to intestinal adaptation.

### 2.2. Small Bowel Bacterial Overgrowth (SBBO)

SBBO contributes to mucosal inflammation, increased intestinal permeability, villus atrophy, deconjugation of bile acids, malabsorption, B12 deficiency, feeding intolerance, bacterial translocation, sepsis, D-lactic acidosis and intestinal failure-associated liver disease (IFALD) [1,13,17,18,19]. SBBO, and associated enteritis, may negatively impact bowel adaptation and ability to wean from PN [17,19].

### 2.3. Blood Stream Infection

Recurrent blood stream infections are common in SBS, and the incidence is seven-times higher in the presence of SBBO [13]. Increased intestinal permeability was reported in three of six paediatric SBS patients with recent episode of sepsis [20]. Catheter-associated infection is increased six-fold in paediatric SBS patients [21], and Gram-negative infections were more common, as compared with non-SBS patients [22]. The increased incidence of sepsis, especially with Gram-negative organisms, in SBS may be due to decreased gut barrier function and increased intestinal permeability in association with SBBO, leading to bacterial translocation.

### 2.4. Intestinal Failure Associated Liver Disease (IFALD)

IFALD is seen in 40%–60% of SBS patients [3,23,24] and is the most common cause of death in these patients [5,6,12,20]. It is a multifactorial disease resulting from the long duration of PN, excess glucose and lipid infusion, components of PN (soya bean lipid; deficiency of essential fatty acids, choline and taurine), sepsis, endotoxins, bowel stasis, lack of enteral feeding, reduced enterohepatic circulation and susceptibility of neonatal liver to cholestatic injury [23,25,26].

### 2.5. Probiotics

Probiotics are live microorganisms, which, when administered in adequate amounts, confer a health benefit on the host. The potential mechanisms by which probiotics may benefit SBS patients include the following.

#### 2.5.1. Role in Gut Maturation and Adaptation

The role of gut commensal organisms in gut maturation was clearly demonstrated in studies of germ-free animals whose intestine was characterised by reduced mucosal cell turnover, enzyme activity, local cytokine production, mucosa-associated lymphoid tissue, lamina propria cellularity, vascularity, muscle wall thickness and motility [27]. Intestinal microbiota have a role in the expression of genes involved in several intestinal functions, including absorption, mucosal barrier function, metabolism, angiogenesis and intestinal maturation [28,29], and probiotics can play this role in enhancing intestinal adaptation in SBS.

Animal studies demonstrate that restoration of healthy microbiota occurs quickly after antibiotic therapy when treated with probiotics [30]. Probiotics, by establishing normal commensals, can aid in the process of gut maturation in SBS infants who are exposed to antibiotics frequently.

Short chain fatty acids (SCFA), resulting from fermentation of carbohydrates and soluble fibre by probiotics, have a trophic role in intestinal adaptation—they reduce ileal mucosal atrophy associated with TPN, increase proliferation and decrease apoptosis of mucosal epithelial cells [31,32,33,34]. *Lactobacillus rhamnosus* GG has been shown to produce soluble proteins that promote growth of intestinal epithelial cells and prevent cytokine-induced apoptosis [35].

#### 2.5.2. Enhancement of Gut Barrier Function

Pathogenic bacteria can increase intestinal permeability by alteration of tight junctions [36], which, combined with abnormal mucosal immunity, may lead to increased bacterial translocation and sepsis. Several studies [37,38,39,40,41,42,43] have confirmed the mucosal barrier-enhancing function of probiotics through their adherence to mucosal surfaces, inhibition of attachment of pathogenic bacteria by competing for binding sites [44,45], secretion of factors that enhance barrier integrity, immunomodulatory effects on cells of the immune system, the preservation of gut epithelial tight junctions with improved occludin, claudin [46] and zona occludens protein expression and increased production of mucin [47,48] and cytoprotective heat shock proteins [49] by intestinal epithelial cells.

#### 2.5.3. Suppression of Pathogens

Probiotics offer colonization resistance by competing for nutrients and attachment sites with pathogenic bacteria and production of antimicrobial molecules. The antibacterial effects of probiotics play an important role in controlling SBBO. Intestinal epithelial cell- and Paneth cell-derived antibacterial peptide (defensins) secretion is induced by probiotics or their components [50]. These peptides display antimicrobial activity against a wide variety of bacteria, fungi and viruses. Probiotics, such as *Lactobacilli* and *Bifidobacterium*, can suppress or directly kill pathogenic bacteria [51,52] by production of antibacterial molecules, including SCFA, acetate and lactate, which lower the luminal pH to inhibit the growth of pathogens [53], and bacteriocins, which attack cell membranes of the target bacteria [54]. *Bifidobacterium* has been shown to produce an unidentified non-protein antimicrobial molecule that inhibits *E. coli*, *Klebsiella pneumoniae*, *Yersinia pseudotuberculosis*, *Staphylococcus aureus* and *Salmonella typhimurium* [52]. Antibiotic-associated diarrhoea, which occurs as a result of ablation of the intestinal microbiota and overgrowth of pathogenic bacteria, such as *Clostridium difficile*, can be ameliorated by probiotics by re-establishing commensal bacteria [55]. 

#### 2.5.4. Immune Modulating Effects

*Lactobacilli* and *Bifidobacteria* enhance total and pathogen specific IgA production in intestinal mucosa without producing probiotic-specific IgA [56,57,58]. *Lactobacillus casei* Shirota has been shown to enhance natural killer cell activity [59]. Downregulation of proinflammatory cytokine production in response to bacterial lipopolysaccharide (LPS) in intestine, liver, plasma and lung has been demonstrated with *Lactobacillus rhamnosus* GG (LGG) treatment in rat infants. LPS-induced pre-necrotic changes in intestinal mucosa were partially prevented with LGG [60]. The TLR9 receptor mediates this effect of probiotics by downregulating inflammatory gene activation [61]. The anti-inflammatory effect of probiotics can potentially modulate gut inflammation associated with SBBO in SBS and promote feed tolerance, as well as protect liver from additional injury. 

#### 2.5.5. Effect on IFALD

Animal studies have demonstrated the protective effect of probiotics on liver by attenuation of liver injury in mouse models of sepsis and alcohol-induced liver injury, purportedly due to enhanced intestinal barrier function, decreased bacterial translocation and endotoxin migration to liver [43,62].

**Hypothesis:** Considering their effects on the gut, we hypothesise that probiotics will be beneficial in SBS through better tolerance of enteral feeding and prevention of bacterial overgrowth and sepsis. Our hypothesis is supported by the results of animal studies showing significant reduction in bacterial translocation and the positive effect on the histological features of intestinal adaptation (Table 1) [63,64,65,66,67,68].

**Aim:** We aimed to conduct a systematic review of studies evaluating probiotic therapy in children with SBS. 

**Methods:** The standard Cochrane methodology [69] was used for this systematic review (Table 2).

**Figure 1:**
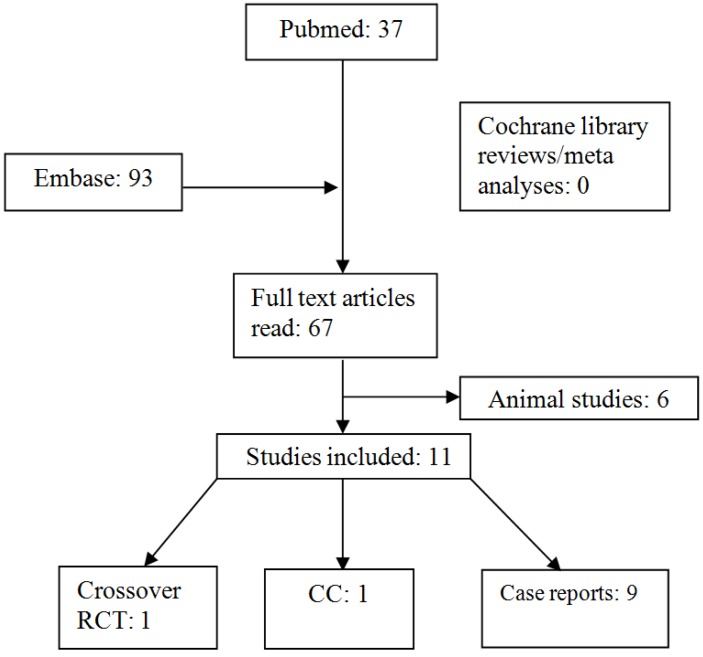
Flow chart of study selection, CC: Case Control study.

**Search Strategy:** The databases, Pubmed, EMBASE and CENTRAL, were searched using the terminologies/MeSH terms “short bowel syndrome” AND Bifidobacterium OR Lactobacillus OR probiotic agent OR probiotics. The international trial registry [70], and the Australian Clinical Trials registry were checked for ongoing/registered trials in this area. No restrictions were applied on study design and language. The search strategy and results are summarised in Table 3 and Figure 1, respectively.

**Table 1:** Experimental studies in animal models of short bowel syndrome (SBS) investigating the effect of probiotics.

	Animal model used	Probiotic used	Results
Eizaguirre *et al.* [63]	Adult Wistar rats (80% bowel resection)	*Bifidobacterium lactis*	BT rate in SBS group 87% *vs.* 50% in SBS-Probiotic group (*p* < 0.05) (RRR was 0.43)
Garcia-Urkia *et al.* [64]	Adult Wistar rats (80% bowel resection)	*Bifidobacterium lactis*	BT rate in SBS probiotic group 44% *vs.* 93% in non-probiotic group
Mogilner *et al.* [65]	Sprague-Dawley rats (75% bowel resection)	*Lactobacillus* GG	BT to liver (60% *vs.* 40%); BT to peripheral blood (40% *vs.* 20%). SBS-Probiotic rats showed a significant increase in crypt depth in ileum and a mild decrease in apoptotic index in jejunum and ileum
Eizaguirre [66].	Adult Wistar rats (80% bowel resection)	*Bifidobacterium lactis*	BT in probiotic group 35% *vs.* 67% in non-probiotic group. Intestinal epithelial proliferation index and proliferation to apoptosis rate higher in probiotic group
Muftoglu *et al.* [67]	Wistar-Albino rats (75% intestinal resection)	*Lactobacillus acidophilus*, *Bifidobacteria*, *Streptococcus thermophilus*	Intestinal diameter, mitotic index, villus length, crypt depth, goblet cell count and immunohistochemical staining for trophic effect significantly increased in jejunum of the SBS-Probiotic group and insignificant increase in ileum
Eizaguirre *et al.* [68]	Adult Wistar rats (80% bowel resection)	*Bifidobacterium lactis*	BT (*E. coli*) rate of 33% (bacterial culture and PCR) as against a rate of 73% by bacterial culture and 87% by PCR in non-probiotic group

BT: bacterial translocation; PCR: polymerase chain reaction; RRR: Relative risk resuction.

**Table 2:** Criteria for selecting studies for review.

Category	Criteria
Study design	RCT, quasi-RCT
Participants	Infants and children with SBS
Interventions	Oral probiotics of any strain, dose or duration, in any form
Comparisons	Probiotics in conjunction with conventional treatment *vs.* conventional treatment with or without placebo
Outcomes	*Primary*: time to full enteral feeds, duration of parenteral nutrition support, growth parameters (weight, height), survival **
	*Secondary*: episodes SBBO, episodes of enterocolitis, episodes of culture proven sepsis, adverse effects of probiotics

RCT: randomized controlled trial.

**Table 3:** Search strategy on Pubmed and Embase.

Search terminologies	Yield
Pubmed: “Short Bowel Syndrome” [Mesh] AND “Probiotics” [Mesh].	25
Pubmed: “Lactobacillus” [Mesh] AND “Short Bowel Syndrome” [Mesh].	26
Pubmed: “Short Bowel Syndrome” [Mesh] AND “Bifidobacterium” [Mesh].	10
Embase: “Short bowel syndrome” AND “Bifidobacterium OR Lactobacillus OR probiotic agent OR probiotics”	93
Final yield after removing overlapping articles	67

The assessment of risk of bias and heterogeneity in the included studies, data extraction and synthesis and pooling of treatment effects was planned according to the standard Cochrane methodology [69]. If possible, subgroup analyses were for the following comparisons and outcomes: (1) type of probiotic/synbiotic, (2) dosage of probiotic, (3) age at intervention, (4) type of feeding: no enteral feeds *vs.* any amount of enteral feeds and (5) short- *vs.* long-term outcomes. 

## 3. Results

Our search revealed no RCTs/Q-RCTs on the use of probiotics in children with SBS. However, we found one small cross-over RCT, one case control study and nine case reports on the use of probiotics in children with SBS. The nine case reports included five reporting beneficial effects (Table 4) and four reporting adverse affects of probiotics.

**Table 4:** Clinical effects of probiotics in SBS.

	Type of study	Age at start of probiotic therapy	Age at bowel resection	Cause of SBS/Small intestine length	Problem before starting probiotics	Probiotics used	Clinical effects reported
Uchida *et al.* (2007) [71]	Case control study Objective: study immunonutritional effects (prealbumin lymphocyte count); faecal flora, faecal SCFA, weight and height velocity after synbiotic therapy in SBS	(1) 2 year	<1 month	(1) Jejunal atresia, 40 cm	Growth retardation home parenteral nutrition abnormal faecal flora	*Bifidobacterium breve* Yakult *Lactobacillus casei* Shirota galactooligosaccharides	Increased faecal *Bifidobacteria*, total facultative anaerobic bacteria, Enterobacteriaceae and *Lactobacilli* Faecal SCFA levels increased Serum concentrations of pre-albumin increased (*p* < 0.05) Lymphocyte counts significantly increased (*p* < 0.05) Increase in height and weight velocity
Vanderhoof *et al.* (1998) [72]	Case report	(1) 7 year	Infancy	(1) Midgut volvulus	SBBO diarrhoea abdominal distension	*Lactobacillus plantarum* 299V	Improvement in stool consistency, reduction of water content Discontinuation of antibiotics (control of SBBO) Weaning of PN Resolution of arthritis
		(2) 14 year	5 year	(2) Midgut volvulus	SBBO diarrhoea abdominal distension arthritis PN	*Lactobacillus plantarum* 299V *Lactobacillus* GG	
Kanamori *et al*. (2001) [73]	Case report	(1) 2 year	1 day	(1) Gastroschisis, 25 cm	enterocolitis, metabolic acidosis and fever episodes poor growth	*Bifidobacterium breve* Yakult *Lactobacillus casei* Shirota galactooligosaccharides	Increase in intrinsic Bifidobacteria and Lactobacilli Levels of E. coli and Candida decreased Ratio of facultative anaerobic bacteria to total bacteria reduced Metabolic acidosis episodes ceased Improved gut motility Accelerated weight gain Increased serum prealbumin and transferrin Tolerance of normal diet instead of elemental diet
Kanamori *et al.* (2004) [74]	Case series	(1) 1 year 3 month		(1) Hirschsprung’s disease	Refractory enterocolitis in all central venous catheter sepsis abnormal intestinal flora	*Bifidobacterium breve* Yakult *Lactobacillus casei* Shirota galactooligosaccharides	Improved faecal flora: increased intrinsic Bifidobacteria and Lactobacilli Pathogenic bacteria (e.g., MRSA Pseudomonas) suppressed Suppression of Candida (detected in only 2 patients of 4) Facultative anaerobic bacteria to anaerobic bacteria ratio reduced (46.9% *vs.* 5.73%) Significant increase of faecal short chain fatty acids (27.8 *vs.* 65.09 μmol/g) Weaning from TPN in 2 dependent patients Accelerated weight gain in all, except one (bowel length 20 cm) Increase of serum prealbumin (*p* < 0.05) Enterocolitis episodes ceased in 3 Reduction in catheter sepsis episodes
		(2) 1 year 4 month		(2) Refractory enterocolitis, 85 cm	TPN		
		(3) 2 year 2 moth		(3) Malrotation, 15 cm	PN		
		(4) 3 year 4 month		(4) Gastroschisis, 25 cm	TPN		
		(5) 4 year 8 month		(5) Hirschsprung’s disease, 100 cm			
		(6) 7 year		(6) Hirschsprung’s disease, 140 cm			
		(7) 20 year 8 month		(7) Malrotation, 60 cm			
Shiau *et al.* (2007) [75]	Case report	(1) 1 month		(1) Midgut volvulus, 10 cm	Diarrhoea PN	*Lactobacillus acidophilus* Bifidobacterium infanti	Cessation of diarrhoea Weaning from PN
Candy *et al.* (2001) [76]	Case report	(1) 11 month	<1 month	(1) NEC, 60 cm	SBBO diarrhoea abnormal small bowel flora negative Na^+^ balance extremely low urine sodium 8 ± 5 mmol/L	*Lactobacillus casei* Shirota	Decreased stool frequency from 12-day to 4-day Increased urine Na^+^ concentration to 92 ± 20 mmol/L (*p* < 0.001)

SCFA: short chain fatty acids; SBBO: small bowel bacterial overgrowth; PN: parenteral nutrition; NEC: necrotizing enterocolitis; TPN: total parenteral nutrition.

**Crossover RCT:** Sentongo *et al.* [77] used the design of a double-blind, placebo-controlled randomised crossover clinical trial to assess the effects of *Lactobacillus rhamnosus* (LGG) treatment on intestinal permeability (IP) in children with SBS. Baseline IP was measured by the urinary lactose-mannitol ratio in nine children with SBS (cases) and 12 healthy children (controls). The median (range) age of the 21 enrolled children was 4.5 (1.6–16.4) years. SBS patients received LGG or placebo for four weeks, followed by a three-week washout period before treatment was crossed over for another four weeks. IP, quantitative faecal cultures for *Lactobacillus* species and the hydrogen breath test (HBT) were performed during LGG and placebo phases of treatment. Baseline IP (mean ± SD) was comparable in cases with SBS and healthy control: 0.08 ± 0.06 *versus* 0.07 ± 0.05 (*p* = 1.0). IP was significantly correlated with age in controls (*r* = −0.83, *p* = 0.001), but not in children with SBS (*r* = −0.55, *p* = 0.16). Faecal colonization [median (range)] with *Lactobacillus* species did not differ during LGG *versus* placebo treatment (1.4 × 10^9^ (4.0 × 10^5^ to 4.0 × 10^9^) cfu/g) *versus* (6.0 × 10^9^ (1.0 × 10^3^ to 1.0 × 10^1^^0^) CFU/g), respectively; (*p* = 0.83). LGG treatment had no consistent effects on IP (*p* = 0.58) or its relationship with age (*r* = −0.40, *p* = 0.29) and was associated with conversion to positive HBT results in one subject. Considering the inconsistent effects of probiotic treatment, it was concluded that empiric LGG therapy to enhance IP in children with SBS was not justified.

**Case-control study:** Uchida *et al.* [71] have evaluated the changes in immunonutritional parameters before and after treatment with a synbiotic (*Bifidobacterium breve*, *Lactobacillus casei*, galactooligosaccharides) in four children with SBS (cases) and four normal, healthy, age-matched children enrolled as controls. Faecal samples were analysed for bacterial flora and organic acid (OA) contents. Levels of short chain fatty acids (SCFA), such as butyrate, propionate and acetate, increased in one patient, and SCFA/total OA levels increased in three patients. Serum lymphocyte counts and pre-albumin levels increased after commencing synbiotic treatment, reaching a statistically significant level at the ninth month compared to the baseline level. There was an increasing trend in height and weight gain velocity during the study *versus* the baseline period. The faecal bacterial flora improved in SBS patients after synbiotic therapy. 

### 3.1. Case Reports on Clinical Benefits of Probiotics in SBS

(1). Vanderhoof *et al.* [72] have reported the use of probiotics in 2 children with SBBO. In the first child (seven-year-old), within in 2–3 weeks of starting *Lactobacillus plantarum* 299V (10^10^ CFU daily), there was reduction in water content and improvement in consistency of stools. In the second child (11-year-old) who had symptoms of abdominal distension, watery and intermittent bloody stools and arthritis, treatment with *Lactobacillus plantarum* 299V (10^10^ CFU daily) facilitated discontinuation of antibiotics, PN, as well as medication for arthritis. 

(2). Kanamori *et al.* [73] have reported treatment of a two-year-old patient with SBS with *Bifidobacterium breve* Yakult, *Lactobacillus casei* Shirota (>1 × 10^9^ bacilli thrice a day) and galactooligosaccharides (3 gm/day) over a period of two years that resulted in dramatic improvement in intestinal motility and absorptive function. Levels of *E. coli* and *Candida* and the ratio of facultative anaerobic bacteria to total bacteria in the faecal samples, which were very high, decreased after synbiotic therapy. The episodes of fever and metabolic acidosis, thought to be related to SBBO, enterocolitis and catheter sepsis, which occurred prior to synbiotic therapy, ceased. There was improvement in the composition of SCFA, with a decrease in the lactate/non-lactate SCFA ratio and an increase in total SCFAs. Weight gain accelerated, and nutritional markers (serum prealbumin, transferrin, choline esterase) increased.

(3). Kanamori *et al.* [74] have reported seven patients suffering from refractory enterocolitis who were treated with *Bifidobacterium breve* Yakult, *Lactobacillus casei* Shirota (1 × 10^9^ bacilli thrice daily) and galactooligosaccharides, which resulted in improved intestinal flora and enteral feed tolerance, facilitating weaning from PN and accelerated weight gain. There was significant increase in the short chain fatty acid content of stools from an average of 27.8 μmol/g to 65.09 µmol/g (*p* < 0.05). Serum prealbumin levels used as a marker of nutritional status significantly increased (*p* < 0.05). Not only the administered probiotic organisms, but also the number of intrinsic *Bifidobacteria* and *Lactobacilli* increased after probiotic therapy in stool samples. Facultative anaerobes were suppressed, while anaerobic bacteria became the predominant organisms. The ratio of facultative anaerobic bacteria to anaerobic bacteria dropped from an average of 46.9% to 5.73%. Pathogenic organisms, including MRSA, *Pseudomonas* and *Candida*, were eliminated or suppressed. Incidence of enterocolitis and sepsis also decreased. Patients who had enterocolitis (treated with antibiotics) in spite of probiotic therapy continued to show predominance of anaerobic bacteria in stools and accelerated weight gain.

(4). Shiau *et al.* [75] reported treatment of a three-month-old 28-week gestation infant with 10 cm residual bowel length with breast milk and *Lactobacillus acidophilus* and *Bifidobacterium infanti* (1 × 10^9^ bacilli per day) for a period of 10 months, which resulted in cessation of diarrhoea, bacterial enteritis and sepsis episodes and a body weight at the 75th centile and length at the 50th centile at one-year follow-up. 

(5). Candy *et al.* [76] reported a positive effect on sodium absorption after commencement of probiotics in a one-year-old infant with SBS. The SBS resulted from NEC, leading to resection of ileum and colon, followed by jejunorectal anastomosis. On a diet of elemental formula and sodium supplements, the urine Na improved from 8 ± 5 mmol/L to 92 ± 20 mmol/L within days of starting on *Lactobacillus casei* Shirota 1.5 × 10^9^ bacilli thrice daily. Stool frequency reduced from 12 to 4 per day.

### 3.2. Case Reports on Complications of Probiotics in SBS

**Probiotic sepsis:** (1) Kunz *et al*. reported *Lactobacillus* (LGG) sepsis in two infants with SBS receiving the probiotic. The infections were successfully treated with ampicillin [78]. The organism causing sepsis in one of the cases was confirmed as the probiotic strain by DNA fingerprinting using pulsed field gel electrophoresis. The route of access to blood for these organisms was speculated to be via translocation from gut, but the possibility of catheter contamination could not be ruled out. (2) De Groote *et al*. [79] described a case of bacteremia after ingestion of a LGG probiotic tablet in an 11-month-old infant with SBS. They used sequencing of the ribosomal operon region and strain typing with pulsed field electrophoresis of the isolates to show identity between the tablet and bloodstream isolates.

**D-Lactic acidosis:** (3) D-Lactic acidosis was reported in a five-year-old girl with SBS receiving Lactomin (*L. acidophilus*, *L. bulgaricus*, *Streptococcus faecalis* and *S. faecium*) suspected to be caused by *L. acidophilus*, which improved after discontinuation of the probiotics [80]. (4) Ku *et al*. reported the case of a five-year-old boy with SBS who developed recurrent episodes of D-Lactic acidosis while on treatment with a probiotic capsule containing *Lactobacillus acidophilus* and *Bifidobacterium infantis*, which resolved when enteral feeds were interrupted. He also developed further episodes when the formula he was receiving was inadvertently changed to a probiotic supplemented formula containing *Lactobacillus acidophilus* and *Bifidobacterium* spp. [81].

## 4. Discussion

The results of our systematic review indicate that there is a paucity of clinical studies on efficacy of probiotics in SBS. We found no RCTs or Q-RCTs in this field. The literature search identified a few animal studies that reported consistent benefits of probiotics in decreasing bacterial translocation and augmenting histological features of intestinal adaptation in SBS. The crossover RCT reported only a non-clinical parameter, such as IP [77]. The baseline IP of subjects with SBS in this trial was comparable to that of controls, most likely due to exclusion of sick and clinically unstable patients, making the applicability of results to SBS patients with complications, such as SBBO, enterocolitis or intestinal failure, difficult. SBBO is known to predispose to intestinal inflammation, and various disorders involving intestinal inflammation are known to have increased IP. Increased IP has also been reported to be associated with a recent episode of sepsis and severe liver disease in patients with SBS, suggesting increased IP may have a role in predisposition to sepsis in SBS [20]. The very small sample size (*n* = 9) and lack of assessment of clinically important outcomes makes it difficult to agree with the author’s conclusion. Whether the three-week wash-out period was adequate to minimise/avoid carry over effects is also not clear. Moreover, it is also important to note that the effects of probiotics are strain-specific, and benefits by probiotic strains other than LGG cannot be ruled out. Analysis of crossover RCTs using paired data from the first and second period of the treatment is a complex issue [69]. Considering the small sample size and the fact that no such data was available, we did not carry a post-hoc analysis of this trial.

The positive impact of probiotic supplementation on growth (increased weight and growth velocity) and nutrition (increased levels of serum proteins) has been demonstrated in the case-control study by Uchida *et al*. [71] and in the case reports (Kanamori *et al*., Shiau *et al*.) [73,74,75]. It is also noteworthy that probiotics have been found to be effective in treating SBBO, enterocolitis and D-Lactic acidosis where conventional treatment modalities have failed (Vanderhoof *et al.*, Kanamori *et al.*) [72,73,74]. Suppression of pathogenic bacteria/facultative anaerobes and normalization of intestinal flora with increased numbers of *Bifidobacteria*, *Lactobacilli* and other anaerobes has been associated with the resolution of these complications (Kanamori *et al.*) [73,74]. The clinically important outcome of treatment with probiotics is improved tolerance of enteral feeds and weaning from TPN, which has been demonstrated in the case reports by Vanderhoof *et al.*, Kanamori *et al.* and Shiau *et al.* [72,73,74,75]. Improvement in gut motility (Kanamori *et al.*) [73] and intestinal absorption (Kanamori *et al.*, Candy *et al.*) [74,76] could be the underlying mechanisms for tolerance of feeds and improvement in symptoms of malabsorption, such as abdominal distension and diarrhoea. A variety of probiotics were used in these studies. 

Our literature search indicates that the commonly reported complications of probiotic treatment in SBS are probiotic sepsis and D-Lactic acidosis. *Lactobacillus* species were implicated in both these complications. *Lactobacillus* sepsis has also been reported in two debilitated children—one, post-cardiac surgery and the other, with cerebral palsy and gastrojejunostomy, receiving probiotic treatment for antibiotic-associated diarrhoea [82]. The tendency of *Lactobacilli* to cause sepsis has been suggested to be related to their increased adherence ability, which is one of the mechanisms of their action [83]. They are also the more commonly used species for probiotic supplementation. One case of *Bifidobacterium* sepsis has been reported in a neonate operated on for omphalocele, which was treated with ampicillin [84]. It is apparent that patients who are most likely to benefit from probiotics are also the ones susceptible to probiotic sepsis. Overall, the incidence of sepsis related to the common probiotic bacteria (*Lactobacilli* and *Bifidobacteria*) has been found to be minimal and is similar to that caused by commensal strains of these bacteria [85]. However, increased surveillance is warranted in patients with debilitating underlying conditions associated with impaired immunity and gut mucosal integrity who are receiving probiotics. This should include investigations for suspected sepsis episodes with anaerobic cultures and molecular identification of the organism if *Lactobacilli* or *Bifidobacteria* are isolated to confirm their identity with probiotic strain, as well as using appropriate empirical antibiotics covering the probiotic strain. It is prudent to select probiotic strains that do not produce D-lactic for therapy in patients with SBS. *Bifidobacterium breve* and *Lactobacillus casei* are theoretically non-D-lactate producing probiotics and, hence, may be suitable for use in neonates and children with SBS. *Bifidobacterium breve* and *Lactobacillus casei* have been used along with antibiotics to treat a SBS patient with D-Lactic acid encephalopathy, which is believed to have replaced D-lactate producing *Lactobacilli* in the gut with L-lactate producing non-pathogenic flora (Uchida *et al.*) [86]. The adverse effects of probiotics have been reported mostly in immunocompromised and debilitated patients and not in healthy individuals [87]. Infants with SBS have compromised gut barrier function and immunity. Hence, extrapolation of results of safety studies in healthy infants, fed with formula containing probiotics [88] to SBS patients is not possible. 

## 5. Conclusions

In summary, our results indicate that there is a paucity of clinical studies of probiotic supplementation in children with SBS. However, the evidence from animal studies and clinical case reports indicate that probiotics do have a potential for benefit in this population of patients and need evaluation in large RCTs. The safety and efficacy of probiotics in SBS can only be answered by multicentre trials, considering the low incidence of this condition. Killed or inactivated probiotic strains, which intuitively cannot have adverse effects, such as probiotic sepsis, but can exert beneficial effects, like live probiotics [89], should also be evaluated in RCTs in SBS patients.
